# STXBP1 forms amyloid-like aggregates in rat brain and demonstrates amyloid properties in bacterial expression system

**DOI:** 10.1080/19336896.2021.1883980

**Published:** 2021-02-16

**Authors:** A.V. Chirinskaite, V.A. Siniukova, M.E. Velizhanina, J.V. Sopova, T.A. Belashova, S.P. Zadorsky

**Affiliations:** a Department of Genetics and Biotechnology, St. Petersburg State University, St. Petersburg, Russian Federation; b Institute of Translational Biomedicine, St. Petersburg State University, St. Petersburg, Russian Federation; c Vavilov Institute of General Genetics, St. Petersburg Branch, Russian Academy of Sciences, St. Petersburg, Russian Federation; d Laboratory of Signal Regulation, All-Russia Research Institute for Agricultural Microbiology, Pushkin, St. Petersburg, Russian Federation; e Laboratory of Amyloid Biology, St. Petersburg State University, St. Petersburg, Russian Federation

**Keywords:** Proteomic screening, rat, brain, Munc18-1, STXBP1, SNARE, amyloid, functional amyloid

## Abstract

Amyloids are the fibrillar protein aggregates with cross-β structure. Traditionally amyloids were associated with pathology, however, nowadays more data is emerging about functional amyloids playing essential roles in cellular processes. We conducted screening for functional amyloids in rat brain. One of the identified proteins was STXBP1 taking part in vesicular transport and neurotransmitter secretion. Using SDD-AGE and protein fractionation we found out that STXBP1 forms small detergent-insoluble aggregates in rat brain. With immunoprecipitation analysis and C-DAG system, we showed that STXBP1 forms amyloid-like fibrils. Thus, STXBP1 demonstrates amyloid properties in rat brain and in bacterial expression system.

## Introduction

Amyloids are fibrillar protein aggregates, which are formed by intermolecular cross-beta sheets [1]. Amyloids have a number of unique features such as insolubility in ionic detergents at room temperature [2] and Congo Red binding with apple-green birefringence when examining stained samples by polarization microscopy [3]. Amyloidogenic proteins were found in different taxa and may have different functions. Pathogenic amyloids are associated with severe diseases in animals and humans such as Alzheimer disease linked with accumulation of Aβ peptide [4] or Parkinson disease associated with aggregation of α-synuclein [5]. However, nowadays more data is emerging about functional amyloids which are not linked with diseases but play essential roles in cellular processes from the formation of a bacterial biofilm [6,7] to the participation in the melanin synthesis in mammals and in particular in humans [8]. In addition, proteins, demonstrating amyloid properties when overproduced and/or in heterologous system, are involved in the control of long-term memory, as it was shown for *Aplysia californica* (protein CPEB) [9] and *Drosophila melanogaster* (protein Orb2) [10].

Most of the amyloids discovered at the moment have been found almost accidentally. The lack of universal methods for the identification of amyloids for a long time did not allow us to fully assess their distribution and role in nature.

We used our original method of screening for detergent-resistant protein aggregates in the brains of young healthy male rats and identified a number of proteins that are potentially able to form amyloids [11,12]. Here we are focused on one particular protein from this list – syntaxin-binding protein (STXBP1, also known as Munc18-1).

STXBP1 is localized in cytoplasm beneath the plasma membrane, it takes part in vesicular transport and neurotransmitter secretion [13]. The protein is presented in brain in two isoforms produced as a result of an alternative splicing: STXBP1a (67,6 kDa) and STXBP1b (68,7 kDa) [14]. The sequences of protein isoforms are almost identical except for about 20 amino acids at C-terminus (Supplementary Fig. S1). A-isoform is major being expressed in specific nuclei of the brainstem, while b-isoform expression is relatively low/absent in these regions in mouse brain [15]. STXBP1 amino acid sequence is conservative over a wide range of animals from fish to mammals, moreover, the sequence of *Rattus norvegicus* protein is identical to syntaxin-binding human protein (Supplementary Fig. S2); thus, sequence conservatism of the studied protein may emphasize its importance in synaptic transmission in brain.

## Results

**Figure 1.**
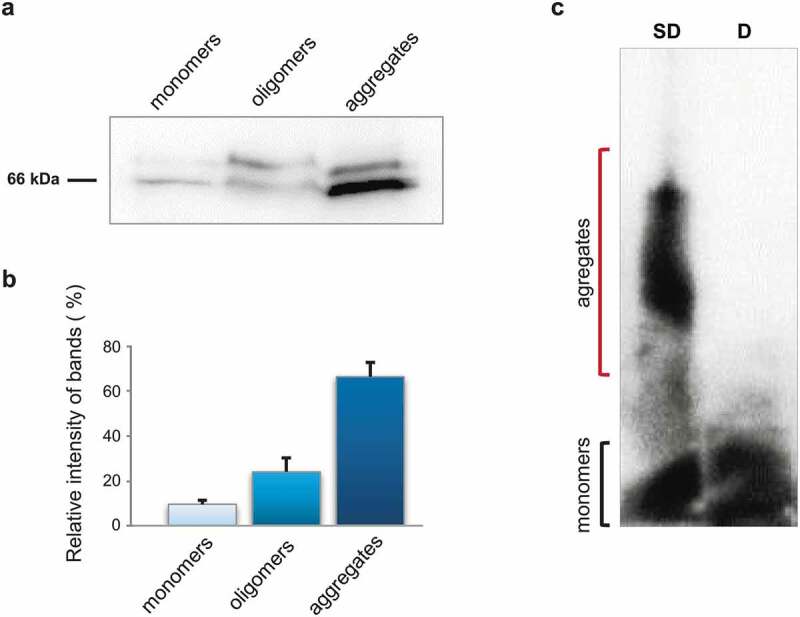
Biochemical analysis of STXBP1 aggregation in rat brain. (a) Size fractionation of rat brain lysate. Total lysate was divided into monomers (less than 100 kDa), oligomers (larger than 100 kDa), and insoluble aggregates. The fractions were separated by SDS-PAGE and analysed by Western-blotting using anti-STXBP1 antibodies. (b) Relative intensity of bands related to STXBP1 monomers, oligomers and aggregates. STXBP is mostly presented in oligomer and polymer fractions. Intensities are represented as mean ± SEM (with three independent brain samples). (c) SDD-AGE of protein lysates extracted from rat brain. Total rat brain lysate was treated with 1% SDS at room temperature (SD) or with 2% SDS at 95°C (d) and subjected to SDD-AGE with subsequent immunoblotting with anti STXBP1 antibodies

To investigate whether STXBP1 is presented in rat brain in aggregated form, total protein lysate from brain was separated into 3 fractions: monomeric fraction less than 100kDa (Figure 1(a)), soluble oligomers and large insoluble aggregates. Via PAGE and subsequent western-blot hybridization using STXBP1-specific antibodies, we showed that the protein is present in all three fractions but mostly in aggregated one (Figure 1(a,b)). Two bands detected in all fractions are corresponding to two known isoforms of STXBP1 [14].

Semi-denaturing detergent agarose gel electrophoresis (SDD-AGE) is a commonly used method for analysing SDS-resistance of protein aggregates based on the insolubility of amyloid aggregates in ionic detergents, such as SDS [16,17]. Total protein lysate from rat brain was treated with 1% SDS and then separated by agarose gel electrophoresis. A significant amount of STXBP1 appears in relatively small detergent-insoluble aggregates (Figure 1(c)). This is consistent with our previous data of proteomic screening for amyloid-forming proteins, where we detected STXBP1 in the fraction of SDS-resistant aggregates [12].

**Figure 2.**
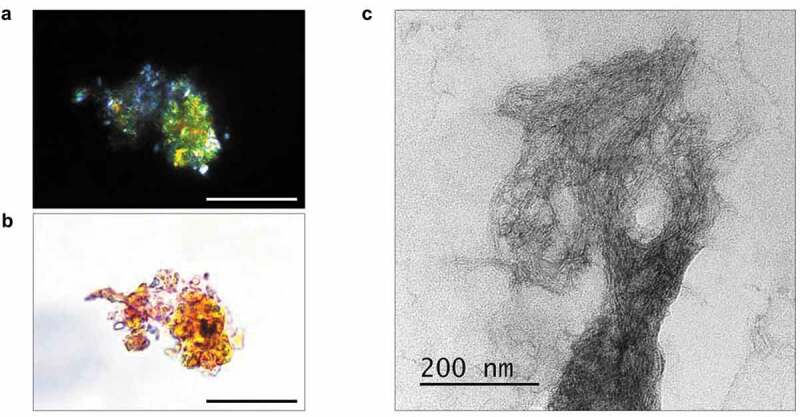
Analysis of amyloid properties of STXBP1 immunoprecipitated from rat brain. The STXBP1 protein which was extracted from rat brain binds Congo red (a) and demonstrates yellow-green birefringence under polarized light (b). Scale bar, 20 μM (c) Fibrils of STXBP1 extracted from rat brain using immunoprecipitation visualized by TEM. Scale bar, 200 nm

Next, we examined amyloid properties of STXBP1 extracted from rat brain using immunoprecipitation with anti-STXBP1 primary antibodies. Isolated protein bound Congo Red and showed apple-green birefringence under polarized light (Figure 2(a,b)). We examined the immunoprecipitated protein using transmission electron microscopy (TEM) and observed fibrils of STXBP1 (Figure 2(c)).

In order to discover STXBP1 regions involved in amyloid aggregates formation in rat brain, we have performed bioinformatic analysis of amino acid sequence using ArchCandy algorithm [18]. We found out that C-terminal parts of both isoforms of the STXBP1 protein contain three potentially amyloidogenic regions: 264–276 a.a., 405–430 a.a. and 548–573 a.a. (Supplementary Fig. S1).

To analyse STXBP1 ability to form amyloid fibrils we used bacterial curli-dependent amyloid generator (C-DAG) system [19,20]. This system relies on the natural ability of *E. coli* cells to produce surface-associated amyloid fibres known as curli, composed mostly of the amyloidogenic protein CsgA. Bipartite N-terminal signal sequence (CsgA_ss_) directs CsgA across the inner and outer membranes to the outside of the cell. Fusion of this signal sequence with the N-terminus of heterologous amyloidogenic protein directs its export to the cell surface, where it assembles into amyloid fibrils. Importantly, protein secretion through the curli export pathway promotes the acquisition of amyloid conformation only for the amyloid-forming proteins [6,21].

Using C-DAG system, we analysed both a and b full-length isoforms of the STXBP1. Besides, we analysed also C-terminal regions of both isoforms (STXBP1a – 252-594 a.a. and STXBP1b – 252-603 a.a.) containing amyloidogenic regions according to ArchCandy data and N-terminal region (1–252 a.a.). The N-terminal region is identical in both isoforms and does nоt contain any regions that can be potentially amyloidogenic. We fused all selected sequences of STXBP1 with CsgA_ss_ and have obtained five plasmids coding for CsgA_ss_-STXBP1a, CsgA_ss_-STXBP1b, CsgA_ss_-STXBP1(1–251), CsgA_ss_-STXBP1a (252–594) and CsgA_ss_-STXBP1b (252–603). All of these chimeric proteins were expressed in the *E. coli* VS39 strain. As controls, we used VS39 *E. coli* transformants with pVS72 and pVS105 plasmids providing the expression of CsgA_ss_-Sup35NM (forms amyloid fibrils, positive control) and CsgA_ss_-Sup35M (does not aggregate, negative control) proteins, respectively.

**Figure 3.**
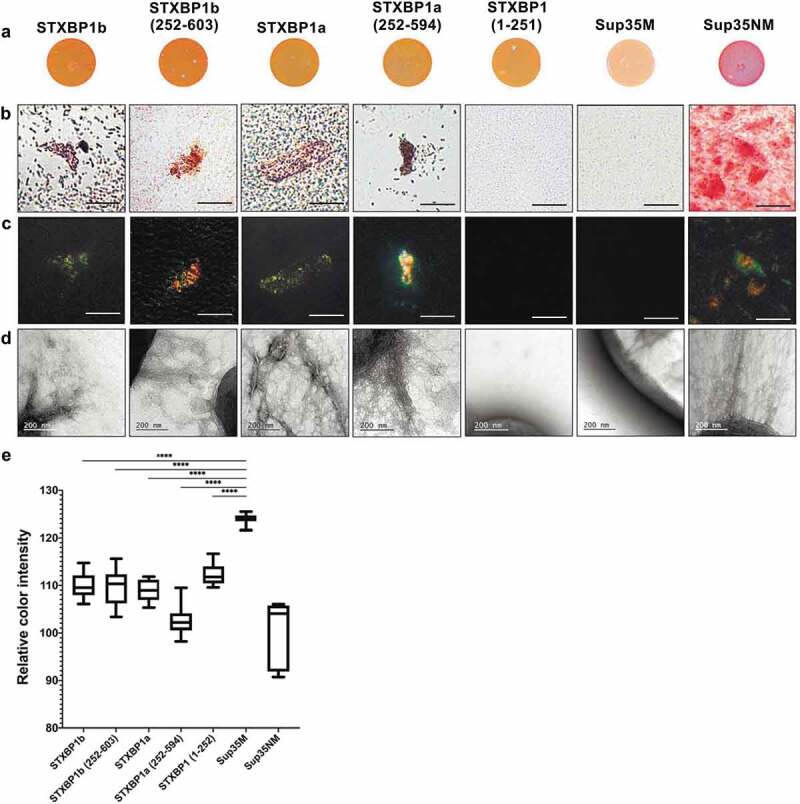
Analysis of amyloid properties of CsgA_ss_-STXBP1a, CsgA_ss_-STXBP1b, CsgA_ss_-STXBP1a(252–594), CsgA_ss_-STXBP1b(252–603), CsgA_ss_-STXBP1(1–251), CsgA_ss_-Sup35M and CsgA_ss_-Sup35NM proteins in C-DAG bacterial system. (a) Colony colour assay of bacteria plated on CR containing medium. CsgA_ss_-Sup35NM so as all CsgA_ss_-STXBP1 forms bind CR. (b, c) Analysis of scraped samples of bacteria grown on CR-comprising medium using polarization microscopy: (b) bright field, (c) crossed polarizers. CsgA_ss_-STXBP1a, CsgA_ss_-STXBP1b, CsgA_ss_-STXBP1a(252–594), CsgA_ss_-STXBP1b(252–603) and CsgA_ss_-Sup35NM bind CR and demonstrate apple-green birefringence when examined between crossed polarizers. Scale bar, 20 μM. (d) Analysis of scraped samples of bacteria using transmission electron microscopy. CsgA_ss_-STXBP1a, CsgA_ss_-STXBP1b, CsgA_ss_-STXBP1a(252–594), CsgA_ss_-STXBP1b(252–603) and CsgA_ss_-Sup35NM form fibrils detectable by TEM. The cultures producing the CsgA_ss_-Sup35NM and CsgA_ss_-Sup35M proteins were taken as positive and negative controls, respectively. (e) Relative colour intensity of bacterial colonies grown on Congo red medium is represented as mean ± SEM. All protein variants are significantly different compared to the negative control. Relative quantification was determined by ImageJ. Statistical analysis was performed using unpaired t test (* p < 0.1, ** p < 0.01, *** p < 0.001, **** p < 0.0001) by Prism

We tested colonies phenotype of all *E. coli* transformants on Congo Red containing medium. We showed that expression of all STXBP1 variants caused red staining of *E. coli* colonies, although the colour was less bright than it was observed in colonies exporting CsgA_ss_-Sup35NM. The colonies exporting CsgA_ss_-Sup35M were pale on the same CR containing medium (Figure 3(a,e)).

Next, we examined Congo-red binding bacteria from the plates using polarization microscopy. We have shown that transformants, expressing both full-length proteins (STXBP1a and STXBP1b), as well as their C-terminal domains (STXBP1a(252–594) and STXBP1b(252–603)) fused to CsgA_ss_, bound Congo Red and exhibit apple-green birefringence when examined between crossed polarizers, similar to Sup35NM (Figure 3(b,c)). This is a typical feature for amyloid fibrils [22]. Despite CR-binding, bacteria expressing CsgA_ss_-STXBP1(1–251) protein did not show apple-green birefringence, neither did CsgA_ss_-Sup35M-expressing bacteria. The analysis of the extracellular matrix, performed by transmission electron microscopy, showed that the same fragments that demonstrated apple-green birefringence (CsgA_ss_-STXBP1a, CsgA_ss_-STXBP1b, CsgA_ss_-STXBP1a(252–594), CsgA_ss_-STXBP1b(252–603) and CsgA_ss_-Sup35NM) are forming detectable fibrils; CsgA_ss_-STXBP1(1–251) and CsgA_ss_-Sup35M do not aggregate (Figure 3(d)). We can summarize that both full-length isoforms of STXBP1 and their C-terminal regions are forming amyloid fibrils in the C-DAG system.

## Discussion

Amyloid proteins are being actively studied nowadays. More data is emerging about functional amyloids in bacteria, insects and even mammals. Our research is also focused on functional amyloids studying. Based on the previous results of proteomic screening which revealed STXBP1 protein in the fraction of detergent-resistant aggregates [12], we aimed to study amyloid properties of STXBP1.

Syntaxin-binding protein is a part of protein complex which mediates vesicle fusion, in particular docking of synaptic vesicles with the presynaptic membrane in neurons [23]. Particular function of syntaxin-binding protein is still unknown. According to the most popular for now hypothesis, syntaxin-binding protein binds both N- and C-terminal parts of syntaxin which in turn leads to changing conformation of syntaxin preventing its interaction with major SNARE (soluble N-ethylmaleimide-sensitive factor attachment receptor) proteins: VAMP and SNAP25 [24]. That leads to an exocytosis halt. If syntaxin-binding protein does not bind C-terminus of syntaxin it allows membrane fusion and further exocytosis [25]. Syntaxin-binding protein is vital for development of nervous system. Deletion of this single protein in mice leads to a complete loss of neurotransmitter secretion from synaptic vesicles throughout development. However, this does not prevent normal brain assembly, including formation of layered structures, fibre pathways, and morphologically defined synapses. After assembly is completed, neurons undergo apoptosis, leading to massive neurodegeneration [26].

It was also shown that the b-isoform of STXBP1 forms aggregates when overexpressed in the hippocampus cells of the *Mus musculus* [15]. Due to our results syntaxin-binding protein normally can aggregate in brain, moreover these aggregates are resistant to SDS (Figure 1(c)), which is typical for amyloids. Protein, immunoprecipitated from rat brain, also shows amyloid properties, including apple-green birefringence when stained with Congo Red (Figure 2(a,b)).

This can be a reason to consider STXBP1 as a possible candidate for being a functional amyloid in mammalian brain. According to our results (Figures 1,Figures 2) we can assume that, if STXBP1 is indeed a functional amyloid, it performs its functions in oligomeric state. Functional role of STXBP1 oligomers in mammalian brain remains to be elucidated. Supposedly, regulated aggregation and disaggregation of the protein can provide a change in the interaction of STXBP1 with syntaxin, affecting the binding of syntaxin to the SNARE complex proteins.

*Rattus norvegicus* STXBP1 amino acid sequence is completely identical to homologous human syntaxin-binding protein. Moreover, the amino acid sequence of syntaxin binding protein is quite conservative throughout mammals, birds, amphibians and even fish (Sup. Fig. S2) which might imply that neurotransmitter release mechanism and in particular it’s protein machinery is versatile and demands strict regulation. Thus, decreased expression level of syntaxin-binding protein in human brain may cause different disorders and diseases. It has been shown that some missense mutations in this protein cause Early Infantile Epileptic Encephalopathy (EIEE), or Ohtahara syndrome, which is one of the most severe forms of age-related epileptic encephalopathies [27]. Some mutations in syntaxin-binding protein gene can cause severe mental retardation or nonsyndromic epilepsy [28]. Overexpression of the mammalian homolog of the STXBP1 has been described in the brain of subjects with schizophrenia [29].

It was shown that mutant syntaxin-binding protein forms large polymers that coaggregate wild-type syntaxin-binding protein *in vitro* and in cell culture [30]. Moreover, this mutant protein also forms Lewy body-like structures that contain α-synuclein (α-Syn); the mutant protein binds and coaggregates α-Syn too [30]. Furthermore, it is shown that trehalose, which is effective in inhibiting aggregation of Aβ, reverses the deficits caused by aggregation of mutant STXBP1 *in vitro* and *in vivo* in multiple models [31]. However, amyloid properties of those aggregates were not examined properly. Having this information and result of our screening, we have decided to check whether STXBP1 has the propensity to form amyloid-like structures. Using C-DAG system we have shown that amyloid fibrils are formed only by proteins containing C-terminal part of the protein containing ArchCandy-predicted amyloidogenic regions (by full-length isoforms of the protein or their C-terminal parts). N-terminal region cannot aggregate at all (Figure 3). According to the data above, we can assume that STXBP1 forms amyloid fibrils in the bacterial expression system and C-terminal region of the protein is essential in amyloid structure formation.

STXBP1 is known as an important brain protein and its structure and quantity should be strictly regulated. Changes in production or conformation of this protein lead to negative consequences such as neurological diseases. In our work we have shown that STXBP1 may form amyloid-like aggregates both in C-DAG system and in the brain of a healthy rat. The formation of STXBP1 amyloid conformers might play a significant role in nervous system functioning, since any changes in the level or mode of aggregation lead to different pathologies. It is still unclear whether STXBP1 detergent-resistant fibrillar aggregates are functional *in vivo* but further investigations in this area may be important for understanding the processes of vesicular transport and neurotransmitter secretion or even for therapy choices.

## Materials and methods

We purchased rats (males, six months old) in Rappolovo laboratory animals breeding colony (Saint-Petersburg, Russia). All experiments involving animals were conducted according to the Directive 2010/63/EU of the European Parliament and of the Council of 22 September 2010. Experiments were approved by the Ethical Committee for Animal Research of St. Petersburg State University (conclusion # 131–03-6). Euthanasia of animals was executed as soon as they were delivered to Saint-Petersburg State University.

Brains after extraction were immediately frozen in liquid nitrogen and homogenized using a cryogenic laboratory mill Freezer/Mill 6870 (SPEX SamplePrep). Homogenates were stored at −70°C. Brain homogenates were solubilized in TBS (Tris-buffered saline) (50 mM Tris-HCl, pH 7.6, 150 mM NaCl), supplemented with Complete Protease Inhibitor (Roche). Then, the probes were centrifuged (5 min., 1500 g, +4°C). Debris was disposed. The lysates obtained were used for further investigation.

For fractionation PAGE experiment rat brain lysates were incubated in 1% SDS solution for 1 hour at room temperature and then were centrifuged at 9800 g for 25 min. Insoluble fraction was collected. Supernatant was subsequently fractionized using Amicon Ultra 100 K filter (Merck, Millipore). Proteins were separated in two fractions: less than 100 kDa and more than 100 kDa. The obtained fractions were used for SDS-PAGE. The separated proteins were transferred onto Immobilon-P PVDF membrane (GE Healthcare, USA) with subsequent Western-Blot hybridization with primary STXBP1-specific monoclonal antibodies ab109023 (Abcam, USA) and secondary Anti-Rabbit IgG antibodies a8275 (Sigma). Chemiluminescent antibody detection was performed using the Amersham ECL Prime Western Blotting Detection Reagent (GE Healthcare, USA)

To analyse the presence of detergent-insoluble aggregates in brain, we separated rat brain lysate proteins by Semi-denaturing detergent agarose gel electrophoresis (SDD-AGE) [32] with subsequent western-blot hybridization performed as described above. SDD-AGE was performed using 1.2% agarose gel. Before separation, proteins were treated for 10 min with 1% SDS at room temperature.

STXBP1 immunoprecipitation from rat brain was conducted using anti-STXBP1 primary antibodies ab109023 (Abcam, USA) bound to protein A coupled SileksMagX-Protein A magnetic beads (Sileks, Russia). The beads were incubated for 2 h with rat brain lysate with addition of Complete Protease Inhibitor (Roche). Protein elution was performed according to manufacturer’s protocol. Finally, the sample was concentrated by ultracentrifugation at 130,000 g for 2 hours.

As a matrix for amplification of DNA fragments coding STXBP1 protein we used pDONR221-Munc18-1 (DNASU Plasmid Repository) for STXBP1a isoform and pAL2-Munc18b (252–603) (Eurogene, Russia) for STXBP1b isoform.

The STXBP1a cDNA coding full-length protein for expression in C-DAG system (*E. coli*) was amplified using STXBP1a CDAGforward and STXBP1a CDAGreverse primers and inserted into pVS105 vector to obtain the pVS-STXBP1a plasmid. The STXBP1a cDNA coding 252–594 a.a. protein fragment was amplified using STXBP1a (252–594) forward and STXBP1a (252–594) reverse primers and inserted into pVS105 vector to obtain the pVS-STXBP1a (252–594) plasmid. The STXBP1a cDNA coding 1–252 a.a. protein fragment was amplified using STXBP1-Nforward and STXBP1-Nreverse primers and inserted into pVS105 vector to obtain the pVS-STXBP1(1–252) plasmid. The STXBP1b cDNA coding 252–603a.a. protein fragment was amplified using STXBP1b (252–603) forward and STXBP1b (252–603) reverse primers and inserted into pVS105 vector to obtain the pVS-STXBP1b (252–603) plasmid. For obtaining of pVS-STXBP1b plasmid we have used DNA coding C-terminal fragment of STXBP1b from pVS-STXBP1b (252–603) plasmid and inserted it in pVS-STXBP1a plasmid instead of DNA coding C-terminal fragment of STXBP1a. The pVS105 plasmid coding Sup35-NM and pVS72 coding Sup35-M were provided by Ann Hochschild [19].

The analysis of amyloid fibril formation of fragments in the bacteria-based C-DAG system was performed according to protocol described earlier [19,20]. *E. coli* strain VS39 was transformed with the following plasmids: pVS-STXBP1a(252–594) plasmid coding for STXBP1a(252–594) protein fused to CsgA signal sequence (CsgA_ss_); pVS-STXBP1a plasmid coding for CsgA_ss_-STXBP1a(1–594) protein; pVS-STXBP1(1–252) plasmid coding for CsgA_ss_-STXBP1(1–252) protein; pVS-STXBP1b(252–603) plasmid coding for CsgA_ss_-STXBP1b(252–603) protein; pVS-STXBP1b plasmid coding for CsgA_ss_-STXBP1b(1–603) protein. VS39 transformants with pVS72 and pVS105 encoding the CsgA_ss_-Sup35NM and CsgA_ss_-Sup35M proteins were used as positive and negative controls of amyloid generation, respectively.

TEM images were recorded on a Jeol JEM-2100 microscope. Negatively stained samples were prepared on formvar film 300 mesh copper grids (Electron Microscopy Sciences). A 10 μl aliquot of bacterial culture suspension was adsorbed to the formvar film for 1 min., blotted, washed three times with 10 μl of water for 10 s, stained with 10 μl of 1% uranyl acetate for 1 min and dried in air.

10 µl of the bacterial suspension was put onto a glass microscope slide, air-dried and analysed in brightfield and between cross polarizers on the inverted microscope Leica DMI6000 B. Images were acquired using the Leica Application Suite software.

## Supplementary Material

Supplemental Material
