# Supplementary material for: STXBP1 forms amyloid-like aggregates in rat brain and demonstrates amyloid properties in bacterial expression system
Source: Prion. 2021 Feb 16;15(1):29–36. doi: 10.1080/19336896.2021.1883980 (PMC7894455; doi:10.1080/19336896.2021.1883980)
Supplement: Supplemental Material [file KPRN_A_1883980_SM4054.pdf]

STXBP1 forms amyloid-like aggregates in rat brain and demonstrates amyloid properties in bacterial expression system

A.V. Chirinskaite, V.A. Siniukova, M.E. Velizhanina, J.V. Sopova, T.A. Belashova, S.P. Zadorsky.

Supplementary Table S1.

Primers used in this study

STXBP1a CDagforward	AATGCGGCCGCACCCATTGGCCTCAAAGCT
STXBP1a CDagreverse	TATTCTAGATTAACTGCTTATTTCTTCATC
STXBP1a (252-594) forward	ATTGCGGCCGCAGAAAATGATGTATACAA
STXBP1a (252-594) reverse	TAATCTAGATTAACTGCTTATTTCTTCATC
STXBP1-Nforward	AATGCGGCCGCACCCATTGGCCTCAAAGCT
STXBP1-Nreverse	ATTTCTAGATTGTATACATCATTTTCG
STXBP1b(252-603) forward	ATTGCGGCCGCAGAAAATGATGTATACCA
STXBP1b(252-603) reverse	TAATCTAGATCACTCCATTGTTGGAGCCT

Supplementary Figure S1.

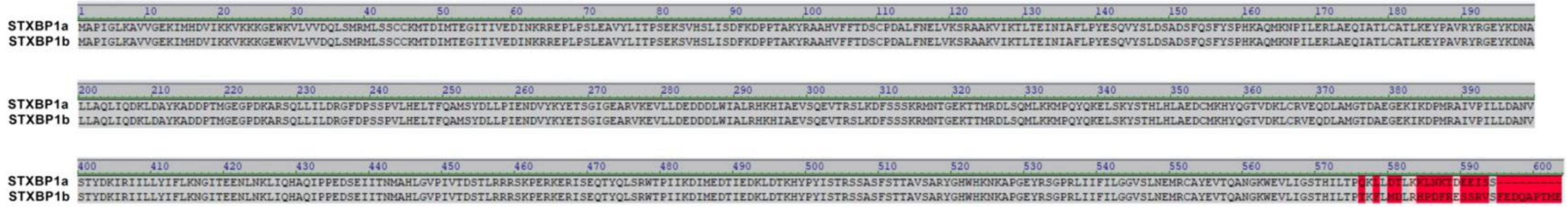

Supplementary Figure S1. Alignment of amino acid sequences of STXBP1 a and b presented in UniProt database (http://www.uniprot.org). Similar amino acids are shown in grey, substitutions for biochemically close amino acids are shown in pink, radical substitution or lack of amino acids are shown in red. The isoforms differ in 20 C-terminal amino acids.

Supplementary Figure S2.

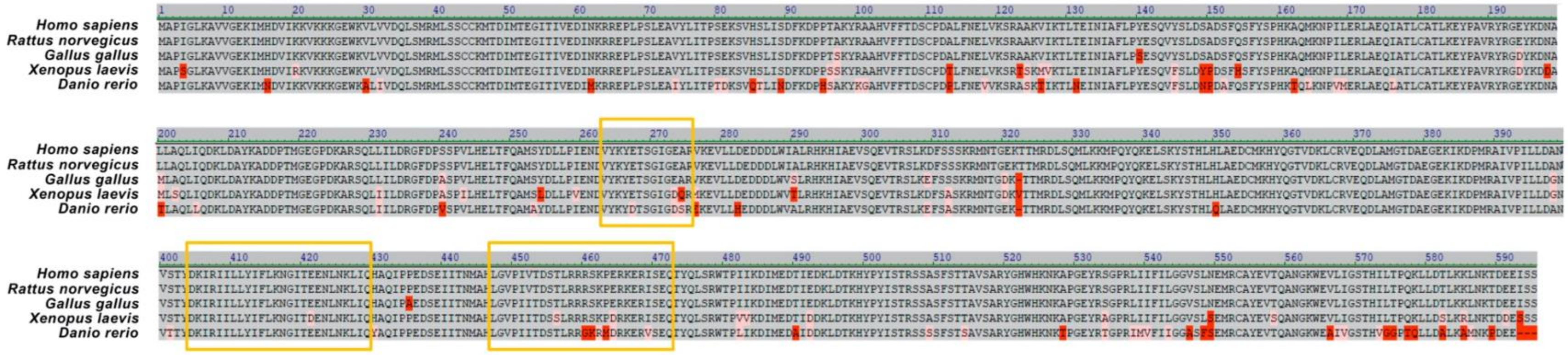

Supplementary Figure S2. Alignment of amino acid sequences of STXBP1 protein of human, rat, bird, frog and fish presented in UniProt database (http://www.uniprot.org). Amino acids similar to human are shown in grey, substitutions for biochemically close amino acids are shown in pink, radical substitution or lack of amino acids are shown in red. Predicted amyloidogenic regions for human and rat are displayed in yellow frames. STXBP1 is highly conservative in warm-blooded animals and shows high similarity generally in vertebrates.
